# A New Homogeneous Catalyst for the Dehydrogenation of Dimethylamine Borane Starting with Ruthenium(III) Acetylacetonate

**DOI:** 10.3390/ma8063155

**Published:** 2015-06-02

**Authors:** Ebru Ünel Barın, Mehdi Masjedi, Saim Özkar

**Affiliations:** Department of Chemistry, Middle East Technical University, 06800 Ankara, Turkey; E-Mails: ebru.unel@metu.edu.tr (E.U.B.); masjedim@missouri.edu (M.M.)

**Keywords:** ruthenium, acetylacetonate, hydrogen generation, dimethylamine borane, dehydrogenation, homogeneous catalysis

## Abstract

The catalytic activity of ruthenium(III) acetylacetonate was investigated for the first time in the dehydrogenation of dimethylamine borane. During catalytic reaction, a new ruthenium(II) species is formed *in situ* from the reduction of ruthenium(III) and characterized using UV-Visible, Fourier transform infrared (FTIR), ^1^H NMR, and mass spectroscopy. The most likely structure suggested for the ruthenium(II) species is *mer*-[Ru(N_2_Me_4_)_3_(acac)H]. Mercury poisoning experiment indicates that the catalytic dehydrogenation of dimethylamine-borane is homogeneous catalysis. The kinetics of the catalytic dehydrogenation of dimethylamine borane starting with Ru(acac)_3_ were studied depending on the catalyst concentration, substrate concentration and temperature. The hydrogen generation was found to be first-order with respect to catalyst concentration and zero-order regarding the substrate concentration. Evaluation of the kinetic data provides the activation parameters for the dehydrogenation reaction: the activation energy *E*_a_ = 85 ± 2 kJ·mol^−1^, the enthalpy of activation ∆*H*^#^ = 82 ± 2 kJ·mol^−1^ and the entropy of activation; ∆*S*^#^ = −85 ± 5 J·mol^−1^·K^−1^. The ruthenium(II) catalyst formed from the reduction of ruthenium(III) acetylacetonate provides 1700 turnovers over 100 hours in hydrogen generation from the dehydrogenation of dimethylamine borane before deactivation at 60 °C.

## 1. Introduction

Catalytic dehydrocoupling of amine-borane adducts has become increasingly important from the perspective of current interest in hydrogen storage [1,2,3], as the efficient storage of hydrogen is still the key issue in hydrogen economy on the way towards a sustainable energy future [4,5]. Of particular importance is the catalytic dehydrogenation of dimethylamine borane (Me_2_HN·BH_3_, DMAB), which can release hydrogen in the presence of suitable catalyst under mild conditions (Equation (1)) [6,7,8,9,10].

(1) $$2\;Me_{2}HN\cdot BH_{3}\;\overset{catalyst}{\to }\;{\left(Me_{2}N\cdot BH_{2}\right)}_{2}+2H_{2}$$

A number of catalysts have recently been employed in dehydrogenation of dimethylamine borane: various Ru, Rh, Pd, and Ir complexes [11], Ru(H)(PMe_3_)(PNP) and *trans*-Ru(H)_2_(PMe_3_)(PNP^H^) [12], [Rh(1,5-cod)(µ-Cl)]_2_ [13,14,15], [Cp_2_Ti] [16,17], [RuH_2_(η^2^-H_2_)_2_(PCy_3_)_2_] and [RuH_2_(η^2^:η^2^-H_2_B-N(Me_2_)_2_(PCy_3_)_2_] [18], RhCl_3_, colloidal Rh/[Oct_4_N]Cl and Rh/Al_2_O_3_ [19], laurate-stabilized Rh(0) [20], hexanoate-stabilized Rh(0) [21], aminopropyltriethoxysilane-stabilized Ru(0) [22], Re complexes [23], Rh_4-6_ clusters [24], RhCl(PHCy_2_)_3_ [25], Ru/ZIF-8 [26], [Ru(p-Cym)(bipy)Cl]Cl [27], Pd(0)/MOF [28], Pd(0)/amylamine [29]. Recent studies have shown that ruthenium(III) acetylacetonate acts as homogeneous catalyst at room temperature in the hydrolysis of sodium borohydride [30,31,32]. This observation prompted us to test the catalytic activity of ruthenium(III) acetylacetonate in the dehydrogenation of DMAB. It was found that the dehydrogenation of DMAB in toluene solution takes place at an appreciable rate in the presence of ruthenium(III) acetylacetonate at temperature above 50 °C and a new ruthenium(II) species is formed during catalytic reaction. The ruthenium(II) species could be characterized by UV-Visible, FTIR, ^1^H NMR, and Mass Spectroscopy. Herein we report the results of catalytic tests using ruthenium(III) acetylacetonate in the dehydrogenation of DMAB in toluene solution as well as the kinetic study of the catalytic reaction depending on the catalyst concentration, substrate concentration and temperature. Our report also includes efforts to identify the catalytically active species in this reaction.

## 2. Results and Discussion

**Figure 1:**
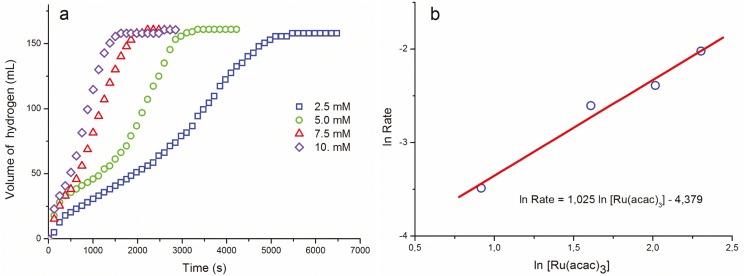
(**a**) Plots of hydrogen volume *versus* time for the dehydrogenation of dimethylamine borane (500 mM) catalyzed by Ru(acac)_3_ as pre-catalyst with different ruthenium concentrations at 60 °C. (**b**) The plot of hydrogen generation rate *versus* the concentration of ruthenium, both in logarithmic scale.

Ruthenium(III) acetylacetonate was found to be a homogeneous catalyst in dehydrogenation of dimethylamine borane at temperatures above 50 °C. The kinetics of the dehydrogenation of dimethylamine borane in the presence of Ru(acac)_3_ were studied by monitoring hydrogen generation depending on catalyst concentration, substrate concentration and temperature. Figure 1a shows the volume of hydrogen *versus* time plots during the dehydrogenation of DMAB solution (500 mM) catalyzed by Ru(acac)_3_ in different ruthenium concentrations at 60.0 ± 0.1 °C.

The inspection of the plots displayed in Figure 1a reveals the following points: (*i*) The increasing catalytic activity observed after a certain period of time (induction time) in each case is indicative of the formation of an active species from the reaction of ruthenium(III) acetylacetonate and dimethylamine borane during the induction time. (*ii*) The active species has higher catalytic activity in comparison to the initial activity observed when the ruthenium(III) acetylacetonate precatalyst was added. (*iii*) The induction time for the formation of active catalyst decreases with the increasing concentration of ruthenium(III) acetylacetonate. (*iv*) Conversely, the catalytic activity during the induction time increases with the increasing concentration of ruthenium(III) acetylacetonate. (*v*) After the induction period, an almost linear hydrogen evolution continues until all the dimethylamine borane is consumed. The rate of hydrogen generation was determined from the slope of the linear portion of plots after the induction time. The hydrogen generation rate increases with the concentration of ruthenium(III) acetylacetonate. The plot of hydrogen generation rate *versus* catalyst concentration, both in logarithmic scale, gives a straight line with a slope of 1.0 as shown in Figure 1b. Thus, hydrogen evolution rate exhibits a first-order dependence on the ruthenium concentration. In other words, the dehydrogenation of dimethylamine borane is first-order with respect to the catalyst concentration.

**Figure 2:**
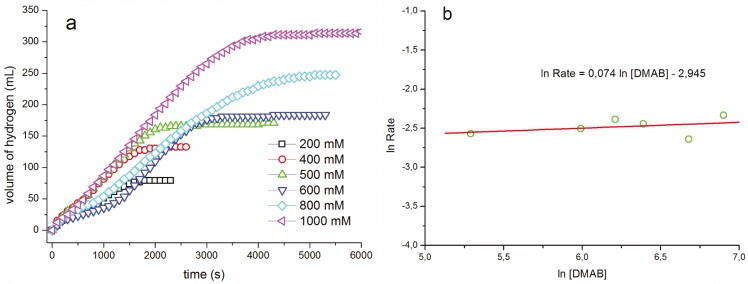
(**a**) Plots of hydrogen volume *versus* time for the dehydrogenation of dimethylamine borane catalyzed by 5.0 mM Ru(acac)_3_ as pre-catalyst with different substrate concentrations at 60.0 ± 0.1 °C. (**b**) The plot of hydrogen generation rate *versus* the concentration of dimethylamine borane, both in logarithmic scales.

The effect of substrate concentration on the hydrogen evolution rate was determined by varying the concentration of NH(CH_3_)_2_BH_3_ at a constant ruthenium concentration of 5.0 mM and temperature of 60.0 ± 0.1 °C (Figure 2a). Figure 2b shows the plots of hydrogen generation rate *versus* NH(CH_3_)_2_BH_3_ concentration, both in logarithmic scale, which gives a horizontal straight line. This indicates that dehydrogenation of dimethylamine borane is zero-order with respect to the substrate concentration, at least in the range of 0.2–1.0 M NH(CH_3_)_2_BH_3_, in the presence of Ru(acac)_3_.

**Figure 3:**
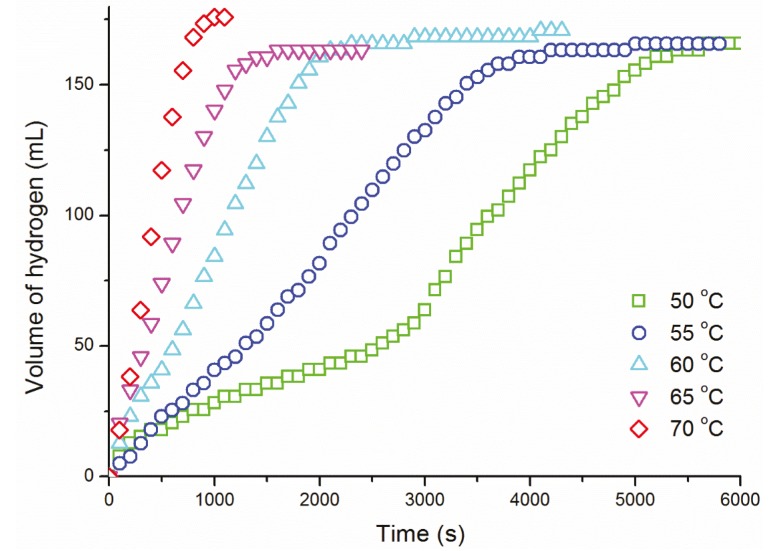
Plots of hydrogen volume *versus* time for dehydrogenation of dimethylamine borane starting with a solution containing 500 mM NH(CH_3_)_2_BH_3_ and 5.0 mM Ru(acac)_3_ at various temperatures.

**Figure 4:**
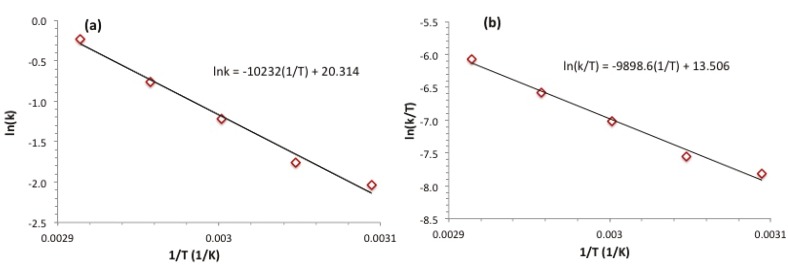
(**a**) Arrhenius plot, (**b**) Eyring plot for the dehydrogenation of dimethylamine borane starting with a solution containing 500 mM NH(CH_3_)_2_BH_3_ and 5.0 mM Ru(acac)_3_ at different temperatures.

Dehydrogenation of dimethylamine-borane was carried out at various temperatures in the range of 50–70 °C starting with a solution containing 500 mM DMAB and 5.0 mM ruthenium(III) acetylacetonate (Figure 3). The examination of the plots given in Figure 3 reveals the following points: (*i*) The induction time for the formation of active catalyst decreases with increasing temperature. (*ii*) The catalytic activity during the induction period and afterwards increases with increasing temperature as the fraction of ruthenium(III) acetylacetonate converted to active catalyst increases concurrently. (*iii*) After the induction time, the values of the rate constant *k* were determined from the linear part of the plots considering the rate dependence on temperature (Table 1) in order to attain the activation parameters for the catalytic dehydrogenation of dimethylamine borane by using either an Arrhenius (Figure 4a) or Eyring plot (Figure 4b). The Arrhenius activation energy was found to be *E*_a_ = 85 ± 2 kJ·mol^−1^ for the catalytic dehydrogenation of dimethylamine-borane starting with Ru(acac)_3_. The Eyring plot provides the activation enthalpy and the activation entropy values: ∆*H*^#^ = 82 ± 2 kJ·mol^−1^ and ∆*S*^#^ = −85 ± 5 J·mol^−1^·K^−1^. The large negative value of activation entropy indicates that the mechanism for the catalytic dehydrogenation of dimethylamine-borane starting with Ru(acac)_3_ has an associative nature in the transition state [33,34].

**Table 1:** Values of the rate constant *k* in (mol H_2_)·(mol Ru)^−1^·s^−1^ for dehydrogenation of dimethylamine borane (500 mM) catalyzed by ruthenium(III) acetylacetonate (5.0 mM) at different temperatures.

*T* (°C)	*k* ((mol H_2_)·(mol Ru)^−1^·s^−1^)
50	1.30 ± 0.07 ×10^−1^
55	1.72 ± 0.09 ×10^−1^
60	2.97 ± 0.15 ×10^−1^
65	4.67 ± 0.23 ×10^−1^
70	7.95 ± 0.40 ×10^−1^

**Figure 5:**
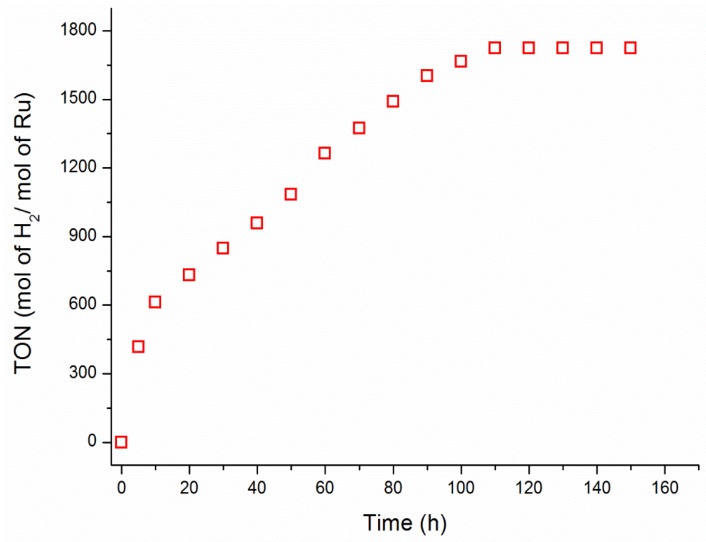
Plot of turnover number *versus* time for the dehydrogenation of dimethylamine borane (3000 mM) catalyzed by Ru(acac)_3_ (5.0 mM) pre-catalyst along with [Ru(N_2_Me_4_)_3_(acac)H] in toluene at 60.0 ± 0.1 °C.

The system comprising Ru(acac)_3_ and NH(CH_3_)_2_BH_3_ appears to be stable and long-lived catalyst in dehydrogenation of dimethylamine borane. The lifetime of the catalyst was measured by determining the total turnover number (TTON) in hydrogen generation from dehydrogenation of dimethylamine-borane at 60.0 ± 0.1 °C. Figure 5 shows the variation in the turnover number with time during the dehydrogenation of dimethylamine borane starting with ruthenium (III) acetylacetonate in toluene at 60.0 ± 0.1 °C. The catalyst formed from the reduction of ruthenium (III) acetylacetonate provides 1700 turnovers over 100 h in the hydrogen generation from the dehydrogenation of dimethylamine borane in toluene at 60.0 ± 0.1 °C. The highest value of turnover frequency (TOF) was found to be 17.8 (mol H_2_)·(mol Ru)^−1^·(min)^−1^ for the catalytic dehydrogenation of dimethylamine borane in toluene at 60.0 ± 0.1 °C.

A mercury poisoning experiment was performed in order to show whether the catalytic dehydrogenation of dimethylamine-borane starting with Ru(acac)_3_ is homogeneous or heterogeneous [35]. The hydrogen generation rate in the system comprising Ru(acac)_3_ precatalyst and *in situ* active catalyst was not affected by the addition of 50 equivalent of mercury to the reaction solution after 50% conversion of NH(CH_3_)_2_BH_3_. This observation indicates unequivocally that the catalytic reaction is homogeneous.

**Figure 6:**
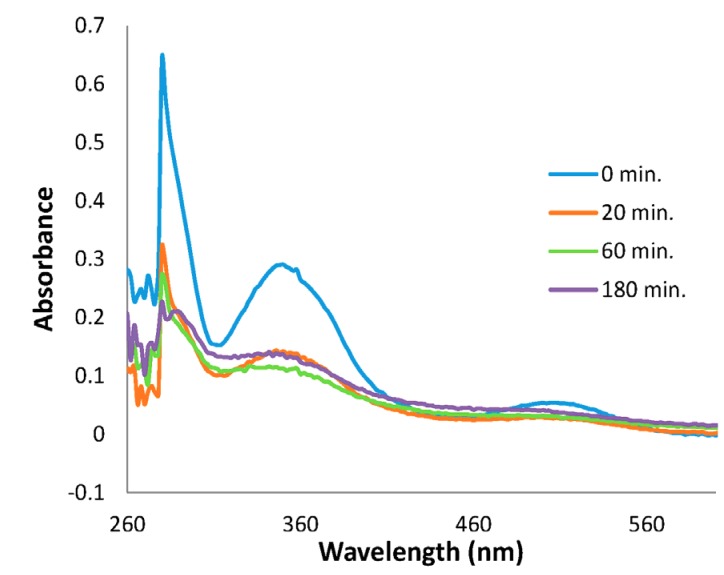
UV-Visible electronic absorption spectra taken from the solution during the catalytic dehydrogenation of dimethylamine borane starting with Ru(acac)_3_.

During the induction, the reaction solution gradually changes its color from red to reddish brown, reflecting the reduction of Ru^3+^. This color change implies that monitoring the UV-vis electronic absorption spectra of the solution may provide a convenient way to follow the conversion. For a better appreciation of the change in the ruthenium complex, the catalytic dehydrogenation of dimethylamine borane starting with ruthenium(III) acetylacetonate was followed by UV-Visible electronic absorption spectroscopy for 3 h even though the reaction is complete in 1 h (Figure 6).

UV-Visible electronic absorption spectrum of sole Ru(acac)_3_ at the beginning of the catalytic reaction shows three prominent absorption bands at 270, 350 and 510 nm. After a certain period of time (induction time), a noticeable increase in the hydrogen generation rate is observed, indicating the formation of a new ruthenium species which has higher catalytic activity in comparison to that of Ru(acac)_3_. As observed in the UV-Visible electronic absorption spectra in Figure 6, a new absorption band at 282 nm grows while the absorption bands of ruthenium(III) disappear. This new absorption feature can be attributed to a ruthenium(II) species, considering its similarity to those reported for octahedral complexes of ruthenium(II), [Ru(en)_2_IP]^2+^ (IP: imidazo[4,5-f][1,10]phenanthroline), [Ru(en)Phen]^2+^ (Phen: 1,10-phenanthroline) [36], and *cis*-[Ru(acac)_2_(P(OMe)_3_)_2_] [37].

**Figure 7:**
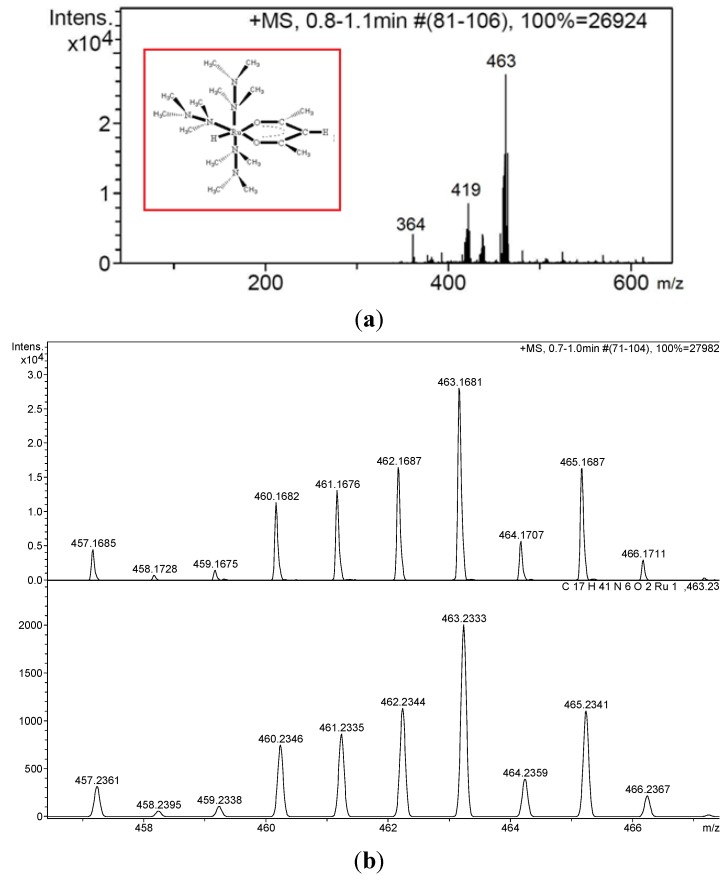
(**a**) Mass spectrum of the ruthenium(II) species, [Ru(N_2_Me_4_)_3_(acac)H], isolated after catalytic dehydrogenation of dimethylamine borane starting with Ru(acac)_3_. (**b**) High resolution mass spectrum of [Ru(N_2_Me_4_)_3_(acac)H] and the isotope distribution simulated for C_17_H_41_N_6_O_2_Ru.

In order to isolate the active catalyst, after the catalytic dehydrogenation of dimethylamine-borane the volatiles were evaporated in vacuum and the residue was dissolved in a mixture of hexane-dichloromethane. Upon cooling the solution to −20 °C, the cyclic dehydrogenation product [(CH_3_)_2_NBH_2_]_2_ of dimethylmine-borane was precipitated out from the solution. After decantation of solution, solvent was removed in vacuum leaving the ruthenium catalyst in the solid residue. Mass spectrum of isolated ruthenium(II) species after the reaction shows a [M-H]^+^ fragment at *m*/*z* = 463 as the base peak (Figure 7a). In addition, two other peaks at *m*/*z* = 419 and *m*/*z* = 364 are observed for the [M–NMe_2_]^+^ and [M-(acac)]^+^ fragments, respectively. The high-resolution mass spectrum in Figure 7b shows the isotope distribution for the molecular peak which matches well with the simulated isotope pattern for the molecular formula C_17_H_41_N_6_O_2_Ru.

The ^1^H NMR spectrum (Figure S1 in the Supplementary Materials), taken from chloroform-d solution, gives a singlet at −5.60 ppm for the hydridic hydrogen directly coordinated to ruthenium. Two other singlets at 1.18 and 0.89 ppm are attributed to the methyl groups of acetylacetonate. A singlet peak is observed at 3.42 ppm for N-Me groups. The FTIR spectrum of the isolated ruthenium(II) species shows absorption bands characteristic of C-H, N-N and C-N stretchings at 2926–2972, 1515 and 1020–1200 cm^−1^, respectively. Accordingly, in the presence of dimethylamine borane as a reducing agent, Ru(acac)_3_ is reduced to a ruthenium(II) species, which is most likely in the form of mer-[Ru(N_2_Me_4_)_3_(acac)H]. The mer-form of the complex is also anticipated to be favorable over the fac-form because of less competition between the O- and N-donor ligand [38,39].

Concerning the formation of [Ru(N_2_Me_4_)_3_(acac)H] complex during the catalytic dehydrogenation of dimethylamine borane starting with ruthenium(III) acetylacetonate, it is likely that dimethylamine borane acts not only as a substrate for releasing hydrogen gas but also as a supplier of tetramethylhydrazine. In addition to the generation of tetramethylhydrazine from the B-N bond dissociation of NH(CH_3_)_2_BH_3_, the formation of instable BH_3_ is also expected which would then be converted to the relatively stable gaseous diborane (B_2_H_6_). The latter gaseous dimer was trapped in the form of trimethylborate in methanol after passing through a bubbler filled with a high-boiling hydrocarbon and identified using an ^11^B NMR spectrum. The observation of a single peak at δ = 16.4 ppm in the ^11^B NMR spectra due to B(OMe)_3_ unambiguously shows the evolution of diborane (B_2_H_6_) during the reduction of ruthenium(III) to ruthenium(II) [40]. There exist precedents in the literature for the formation of tetramethylhydrazine from dimethylamine [41,42]. In particular, the formation of N_2_Me_4_ from dimethylamidotitanium has significant relevance to the present study [43].

It is noteworthy that the isolated ruthenium(II) species, mer-[Ru(N_2_Me_4_)_3_(acac)H], shows a catalytic activity in dehydrogenation of dimethylamine-borane, almost comparable to the initial activity when started with Ru(acac)_3_, but without an induction period as the experiment started with a preformed catalyst. However, we have to admit that measuring the activity has noticeable uncertainty because of the insufficient amount of isolated complex and impurities in it.

## 3. Experimental Section

### 3.1. Materials

Ruthenium(III) acetylacetonate, borane dimethylamine complex (97%), toluene (99.7%) and hexane (99%) were purchased from Sigma-Aldrich (St. Louis, MO, USA). Dichloromethane (99%) was purchased from Merck^®^ (Darmstadt, Germany). All glassware and Teflon coated magnetic stir bars were cleaned with acetone, followed by copious rinsing with distilled water before drying at 150 °C for a few hours.

### 3.2. Equipment

All reactions were performed under argon or nitrogen atmospheres and carried out in a Parr-5101 low pressure stirred reactor with a circulating water-bath for constant temperature control. The Parr-5101 low-pressure stirred reactor was connected with a digital transmitter to a computer using a RS-232 module. The progress of an individual dehydorgenation reaction was followed by monitoring the increase in the pressure of hydrogen gas with the program Calgrafix. The temperature was also controlled via a thermocouple placed inside the reactor. ^1^H NMR spectrum was measured on a Bruker Avance (III) 400 MHz spectrometer, chemical shifts are given in ppm (δ) relative to Me_4_Si as internal standard. UV-visible electronic absorption spectra were recorded on a Schimadzu-2450 double beam spectrometer. The infrared spectrum was recorded from a Vertex 70 ATR/FTIR spectrometer. Positive ion mass spectrometry data was acquired on a Micro TOF-LC/ESI/Ms system.

### 3.3. Catalytic Dehydrogenation of Dimethylamine Borane Starting with Ruthenium(III) Acetylacetonate

In order to test the catalytic activity of ruthenium(III) acetylacetonate in the dehydrogenation of dimethylamine borane, 500 mM dimethylamine borane was dissolved in 10 mL toluene in a reactor thermostated to 60 °C under inert atmosphere. Afterwards, 5 mM Ru(acac)_3_ was added into the reaction solution. After the addition of Ru(acac)_3_, the reactor was closed immediately and stirring at 1000 rpm was turned on. Hydrogen started to evolve from the reaction and increase pressure inside the reactor. Pressure inside the reactor was recorded every 5 seconds. All reactions were performed in 10 mL of toluene in which dimethylamine borane (within the range of 200–1000 mM) and Ru(acac)_3_ (within the range of 2.5–5.0 mM) are dissolved. Kinetics of dehydrogenation of dimethylamine borane catalyzed by Ru(acac)_3_ as pre-catalyst was studied depending on substrate concentration, catalyst concentration and temperature. In a set of experiments, NH(CH_3_)_2_BH_3_ concentration was held constant at 500 mM and Ru(acac)_3_ concentration was varied in the range of 2.5, 5.0, 7.5 and 10 mM at 60.0 ± 0.1 °C. In the second set of experiments, Ru(acac)_3_ concentration was held constant at 5 mM, while NH(CH_3_)_2_BH_3_ concentration was varied in the range of 200, 400, 500, 600, 800 and 1000 mM at 60.0 ± 0.1 °C. The third set of experiments were performed by keeping NH(CH_3_)_2_BH_3_ and Ru(acac)_3_ concentrations constant at 500 and 5 mM, respectively, and varying the temperature in the range of 50, 55, 60, 65 and 70 °C in order to obtain the activation energy (*E*_a_), enthalpy of activation (∆*H*^#^) and entropy of activation (∆*S*^#^). In all sets of experiments, the hydrogen evolved from the dehydrogenation reaction was followed by monitoring the increase in the pressure of hydrogen gas with the program Calgrafix. The pressure *versus* time data was processed using Microsoft Office Excel 2003 and Origin 7.0 and then converted into the values in the proper unit, volume of hydrogen (mL).

### 3.4. Catalytic Lifetime of Ruthenium(III) Acetylacetonate

The catalytic lifetime of Ru(acac)_3_ in dehydrogenation of dimethylamine borane was determined by calculating the total turnover number (TTO). The life time experiment was started with 10 mL toluene solution containing 5 mM Ru(acac)_3_ and 3000 mM NH(CH_3_)_2_BH_3_. Hydrogen gas started to evolve from the reaction. When 75 percent of hydrogen generation was achieved, more NH(CH_3_)_2_BH_3_ was added to the reaction solution. By this way, the addition of NH(CH_3_)_2_BH_3_ was repeated until no more hydrogen gas was evolved. The TTO, that is, the number of moles of hydrogen evolved per number of moles of ruthenium, was calculated.

### 3.5. Poisoning Experiment

In order to understand whether dehydrogenation of dimethylamine borane catalyzed by Ru(acac)_3_ is homogenous or heterogeneous, mercury poisoning experiment was carried out [44]. After the 50% conversion of reaction was attained, 50 equivalent of mercury per ruthenium was added to the reaction solution including 5 mM Ru(acac)_3_ and 500 mM NH(CH_3_)_2_BH_3_.

### 3.6. Isolation and Characterization of Rutneium(II) Species, [Ru(N_2_Me_4_)_3_(acac)H]

19.92 mg Ru(acac)_3_ (5 mM) was added into the reaction flask containing 294.6 mg NH(CH_3_)_2_BH_3_ (500 mM) dissolved in 10 mL toluene and thermostated at 60.0 ± 0.1 °C. Although the complete conversion took 1 hour, the reaction was carried on for 3 h under inert atmosphere and followed by UV-Vis spectroscopy. After 3 h stirring, the solvent was evaporated in vacuum and the residue was dissolved in the mixture of hexane-dichloromethane. Then, the mixture was put in a fridge in order to precipitate out NH(CH_3_)_2_BH_3_ and the cyclic product formed during dehydrogenation of NH(CH_3_)_2_BH_3_. At the next step, the solution was filtered and evaporated in vacuum giving about 8 mg [Ru(N_2_Me_4_)_3_(acac)H] complex (35% yield). [Ru(N_2_Me_4_)_3_(acac)H]: ^1^H NMR (CDCl_3_, ppm): δ-5.60 (br s, 1H, Ru-H), 0.89 (br s, 3H, CH_3_), 1.18 (s, 3H, CH_3_), 3.42 (br s, 36H, N-CH_3_). FTIR (neat, n, cm^−1^): 2972 m, 2926 m, 1515 m, 1350–1470 s, 1020–1200 m. UV: λ_max_ (Toluene, nm) 282. Mass: *m*/*z* 463 ([M-H]^+^, 100%), 419 (32), 364 (15). A ^13^C NMR spectrum couldn’t be taken because there was an insufficient amount of sample.

## 4. Conclusions

In summary, our study on the catalytic dehydrogenation of dimethylamine-borane starting with ruthenium(III) acetylacetonate produced the following conclusions and insights, some of which were previously unavailable: (*i*) Ru(acac)_3_ is a pre-catalyst in dehydrogenation of dimethylamine-borane, being converted to an active species. (*ii*) A new ruthenium(II) species is formed during the catalytic dehydrogenation of dimethylamine-borane starting with Ru(acac)_3_ after the induction time and isolated in the form of *mer*-[Ru(N_2_Me_4_)_3_(acac)H]. (*iii*) Increasing the concentration of Ru(acac)_3_ and applying a higher temperature leads to a decrease in the induction time and an increase in the formation rate of active catalyst. (*iv*) The new ruthenium(II) complex, *mer*-[Ru(N_2_Me_4_)_3_(acac)H], isolated from the catalytic reaction solution, provides faster dehydrogenation of dimethylamine-borane than that obtained initially by starting with Ru(acac)_3_. (*v*) Dehydrogenation of dimethylamine-borane starting with ruthenium(III) acetylacetonate is first-order with respect to the catalyst concentration and zero-order with respect to substrate concentration. (*vi*) The catalyst formed from Ru(acac)_3_ provides 1700 turnovers over 100 h in hydrogen generation from the dehydrogenation of dimethylamine-borane before its deactivation. (*vii*) A UV-Visible electronic absorption spectrum of the reaction solution after induction period remains unaltered during the whole catalytic reaction and is the same as that of the isolated *mer*-[Ru(N_2_Me_4_)_3_(acac)H] complex. This observation supports the hypothesis that *mer*-[Ru(N_2_Me_4_)_3_(acac)H] isolated from the reaction solution is the active catalyst rather than its conversion product. (*viii*) The activation parameters of catalytic dehydrogenation of dimethylamine-borane starting with ruthenium(III) acetylacetonate, determined from the evaluation of the kinetic data, are activation energy *E*_a_ = 85 ± 2 kJ·mol^−1^, enthalpy of activation ∆*H*^#^ = 82 ± 2 kJ·mol^−1^ and entropy of activation ∆*S*^#^ = −85 ± 5 J·mol^−1^·K^−1^.

## Supplementary Materials

Supplementary materials can be accessed at: http://www.mdpi.com/1996-1944/8/6/3155/s1.
