# Supplementary material for: A New Homogeneous Catalyst for the Dehydrogenation of Dimethylamine Borane Starting with Ruthenium(III) Acetylacetonate
Source: Materials (Basel). 2015 Jun 2;8(6):3155–67. doi: 10.3390/ma8063155 (PMC5455753; doi:10.3390/ma8063155)
Supplement: Supplementary file 1 [file materials-08-03155-s001.pdf]

Supplementary Materials

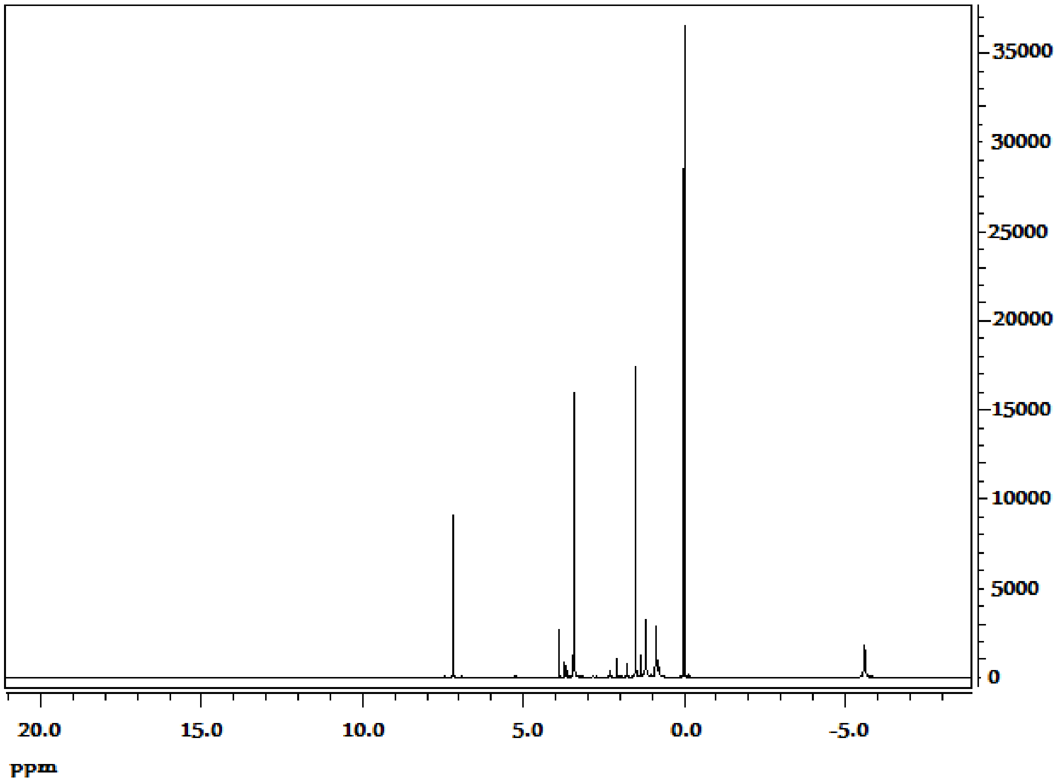

Figure S1. The ^{1}H NMR spectrum of the ruthenium(II) species, $[Ru(N_2Me_4)_3(acac)H]$, isolated after catalytic dehydrogenation of dimethylamine borane starting with $Ru(acac)_3$, taken from chloroform-d solution.
